# Community Mental Health Clinicians’ Perspectives on Telehealth During the COVID-19 Pandemic: Mixed Methods Study

**DOI:** 10.2196/29250

**Published:** 2022-03-03

**Authors:** Simone H Schriger, Melanie R Klein, Briana S Last, Sara Fernandez-Marcote, Natalie Dallard, Bryanna Jones, Rinad S Beidas

**Affiliations:** 1 Department of Psychology University of Pennsylvania Philadelphia, PA United States; 2 Department of Psychiatry University of Pennsylvania Philadelphia, PA United States; 3 Community Behavioral Health Philadelphia, PA United States; 4 Department of Medical Ethics and Health Policy Perelman School of Medicine University of Pennsylvania Philadelphia, PA United States; 5 Department of Medicine Perelman School of Medicine University of Pennsylvania Philadelphia, PA United States; 6 Penn Implementation Science Center at the Leonard Davis Institute of Health Economics University of Pennsylvania Philadelphia, PA United States; 7 Penn Medicine Nudge Unit University of Pennsylvania Health System Philadelphia, PA United States; 8 Center for Health Incentives and Behavioral Economics University of Pennsylvania Philadelphia, PA United States

**Keywords:** telehealth, COVID-19, evidence-based practice, community mental health, trauma-focused cognitive behavioral therapy, implementation science, youth mental health

## Abstract

**Background:**

In March 2020, a rapid shift to telehealth occurred in community mental health settings in response to the need for physical distancing to decrease transmission of the virus causing COVID-19. Whereas treatment delivered over telehealth was previously utilized sparingly in community settings, it quickly became the primary mode of treatment delivery for the vast majority of clinicians, many of whom had little time to prepare for this shift and limited to no experience using telehealth. Little is known about community mental health clinicians’ experiences using telehealth. Although telehealth may make mental health treatment more accessible for some clients, it may create additional barriers for others given the high rates of poverty among individuals seeking treatment from community mental health centers.

**Objective:**

We examined community mental health clinicians’ perspectives on using telehealth to deliver trauma-focused cognitive behavioral therapy to youth. We sought to better understand the acceptability of using telehealth, as well as barriers and facilitators to usage.

**Methods:**

We surveyed 45 clinicians across 15 community clinics in Philadelphia. Clinicians rated their satisfaction with telehealth using a quantitative scale and shared their perspectives on telehealth in response to open-ended questions. Therapists’ responses were coded using an open-coding approach wherein coders generated domains, themes, and subthemes.

**Results:**

Clinicians rated telehealth relatively positively on the quantitative survey, expressing overall satisfaction with their current use of telehealth during the pandemic, and endorsing telehealth as a helpful mode of connecting with clients. Responses to open-ended questions fell into five domains. Clinicians noted that (1) telehealth affects the content (ie, what is discussed) and process (ie, how it is discussed) of therapy; (2) telehealth alters engagement, retention, and attendance; (3) technology is a crucial component of utilizing telehealth; (4) training, resources, and support are needed to facilitate telehealth usage; and (5) the barriers, facilitators, and level of acceptability of telehealth differ across individual clinicians and clients.

**Conclusions:**

First, telehealth is likely a better fit for some clients and clinicians than others, and attention should be given to better understanding who is most likely to succeed using this modality. Second, although telehealth increased convenience and accessibility of treatment, clinicians noted that across the board, it was difficult to engage clients (eg, young clients were easily distracted), and further work is needed to identify better telehealth engagement strategies. Third, for many clients, the telehealth modality may actually create an additional barrier to care, as children from families living in poverty may not have the requisite devices or quality broadband connection to make telehealth workable. Better strategies to address disparities in access to and quality of digital technologies are needed to render telehealth an equitable option for all youth seeking mental health services.

## Introduction

The public health response to the COVID-19 pandemic has resulted in a rapid transformation in mental health care delivery of psychosocial treatments. Within a matter of days in the spring of 2020, clinicians went from primarily treating clients in their offices to almost exclusively treating clients via telehealth (ie, telecommunications platforms through which mental health treatments can be delivered; also referred to as telemental therapy, virtual therapy, and teletherapy). Although real-time (ie, not prerecorded) clinician delivery of evidence-based practices (EBPs) using telehealth are effective for several mental health conditions for adults (eg, anxiety disorders, depression, obsessive-compulsive disorder [OCD], panic disorder, posttraumatic stress disorder [PTSD], psychotic disorders) and children (eg, depression, OCD, PTSD, tic disorders), this treatment modality was not widely adopted by US health systems prior to the pandemic [1-15].

In light of this rapid transformation, the American Psychological Association (APA) conducted surveys of its members to understand telehealth use prior to and after the pandemic, which captured this surge in use. Prior to 2020, only 1% of clinicians saw clients exclusively over telehealth and only 20% used telehealth with any of their clients [16,17]. Within 3 months of the start of the pandemic, 92% of clinicians were seeing clients over telehealth, and within 6 months, this proportion had risen to 96%. Even the Veteran’s Health Administration system, which had a robust telehealth system prior to the pandemic, increased their telemedicine use by 12-fold [18], with over 1 million mental health telephone and video encounters with veterans occurring in a single month of the pandemic [19]. The unprecedented shift toward telehealth has been made possible in large part due to the temporary lifting of regulatory barriers such as billing, insurance coverage, insurance reimbursement, and licensure regulations that limit treatment of clients across state lines [20]. For example, in the United States, emergency mandates and policies allow for temporary reimbursement of remote mental health services and for practice across state lines (eg, a clinician licensed in Pennsylvania can see a client located in New Jersey). The uptake in the use of telehealth has been accompanied by expansions in telehealth infrastructure and advances in telehealth technology, which are ongoing [21]. Some have suggested that even when the pandemic is over and the state of emergency has passed, a full return to in-person services is unlikely [22].

Telehealth is often described as a tool to reduce barriers to care for clients who may otherwise have difficulty attending appointments. For some clients, however, telehealth and technology-based services may actually exacerbate existing socioeconomic disparities, particularly within low-income populations such as those seeking treatment in community mental health (CMH) settings [23]. Digital disparities, including differential access to technological devices and quality internet, and disparities in technological literacy, may exacerbate inequities in access to mental health treatment. Although 75% of US adults have access to broadband internet in their homes, the 25% who lack access have disproportionately lower income and education, and are more likely to be racial minorities, older, rural residents, widowed, and living with disabilities [24]. Given the sociodemographic characteristics of clients who seek care in CMH settings, inequitable care is an urgent concern, particularly because reliable access to the internet has been characterized as a “super-determinant” of health [25]. A recent study found that within a sample of Medicare beneficiaries, around 40% lacked access to either a computer with high-speed internet or a smartphone with a data plan and 26% lacked access to both, and these patterns of inequity exist for children as well [26,27]. This level of access may be even lower among Medicaid beneficiaries.

These access issues are particularly important to address in the context of the COVID-19 pandemic, in response to which there is a rising need for mental health care. The rise in telehealth use during the pandemic is likely due not only to safety issues associated with in-person care (due to needs for physical distancing to decrease transmission of the virus) but also to the increased incidence of mental disorders such as anxiety and depression [28]. In addition to the increase in common mental disorders, the changes brought about by the pandemic (such as stay-at-home orders) have also been associated with an increase in traumatic incidents, many of which involve or are witnessed by youth [29]. Thus, there is a need for delivery of trauma-informed EBPs to youth, particularly in CMH settings, where baseline rates of trauma in youth seeking treatment are high. One EBP that has been widely rolled out in the United States, trauma-focused cognitive behavioral therapy (TF-CBT), is particularly relevant. TF-CBT is an evidence-based, short-term intervention for youth exposed to trauma that typically involves weekly 60-minute sessions delivered over the course of 12-25 sessions and involves participation of caregivers [30]. TF-CBT involves collaboration among the clinician, client, and caregiver to provide the client with psychoeducation and skills in a supportive environment through which the client can process traumatic memories. TF-CBT, the core components of which are often referred to with the acronym “PRACTICE,” includes psychoeducation and parenting skills, relaxation techniques, and support in affective expression, among other skills. Central to the intervention is the use of exposure to upsetting memories (via the trauma narrative) and exposure to trauma reminders (via in vivo exposure) to support clients in becoming less fearful. Studies suggest that TF-CBT can be effectively delivered via telehealth with children at school and at home [14]. However, certain adaptations to TF-CBT are needed, particularly to components that fall within the “adaptable periphery” of the intervention (ie, elements of an intervention or how it is delivered that can be altered while maintaining treatment fidelity) rather than the “core components” (ie, the crucial and unexchangeable elements of the intervention that underlie its effectiveness) [31-33]. Particular consideration must be given to the trauma narrative, a core exposure-based treatment element wherein the client creates and shares a narrative of the traumatic event that occurred. Although the trauma narrative can take multiple forms, clients typically choose to create a written narrative. In the context of telehealth, clients may dictate the trauma narrative while the clinician types it and shares their screen. Clinicians have documented their adaptations to exposure-based treatments using telehealth [34].

Even when adaptations are made to facilitate delivery of TF-CBT via telehealth, additional challenges remain [35,36]. For example, clients may have attention difficulties that impair their ability to engage in therapy, or may struggle to secure a safe and private place to engage in therapy at home or school. These challenges may impair their ability to speak freely and may necessitate shorter sessions. Additionally, children and the adults supervising them may not have the skills to easily navigate digital platforms. The virtual format also presents particular challenges to the delivery of the trauma narrative. For example, clinicians may find it challenging to read clients’ body language and affect or to identify their dissociation over the screen, making it difficult to assess clients’ level of distress and to modulate exposure potency accordingly. Clinicians may also find it challenging to help clients regulate their affect via the telehealth format.

Although previous studies have illuminated some of the challenges associated with delivery of TF-CBT via telehealth, these studies were carried out in an academic medical setting and within a context that provided clients with access to technological devices and internet connectivity. Studies on clinician satisfaction with and ratings of telehealth acceptability have largely been conducted in private systems, which may differ substantially from community clinics given the higher resources, lower caseloads, and less complex clients compared with those of public systems [37]. Thus, little is known about CMH clinicians’ perspectives on delivering EBPs through a virtual platform within the context of usual care.

In this study, we focused on clinician perspectives on telehealth delivery of TF-CBT with a CMH population within the first 6 months of the COVID-19 pandemic. Given the widespread implementation of TF-CBT nationally and the similarity in implementation barriers faced across EBPs and across CMH settings, we view this study as a potential exemplar for understanding how clinicians delivering EBPs in CMH settings more broadly experienced this shift to telehealth [38-40]. We also view this survey as an opportunity to identify and highlight considerations for future directions in improving and sustaining telehealth delivery within the CMH context.

## Methods

### Study Setting

This study was conducted in the Community Behavioral Health (CBH) network of public behavioral health clinics in Philadelphia. CBH is a not-for-profit contracting organization that serves as the exclusive payer for Medicaid-funded services in Philadelphia, and has supported the implementation of EBPs in CMH organizations since 2007. As a response to high rates of youth trauma exposure, the Philadelphia Department of Behavioral Health and Intellectual Disability Services (DBHIDS) developed a comprehensive trauma-informed public behavioral health system in 2011, and in 2012, DBHIDS was awarded a National Child Traumatic Stress Initiative Community Treatment and Service Center grant to form the Philadelphia Alliance for Child Trauma Services (PACTS). This initiative is centered around increasing the number of children in Philadelphia receiving evidence-based trauma treatment such as TF-CBT [41]. To date, PACTS has trained 11 cohorts of clinicians in TF-CBT across both outpatient and residential CMH agencies.

### Study Procedures

Study procedures were approved by the City of Philadelphia Department of Public Health and the University of Pennsylvania Institutional Review Boards. As part of a survey to broadly assess TF-CBT clinicians’ experiences during the first 6 months of the COVID-19 pandemic, all clinicians who had been trained through the PACTS initiative (N=198) and were currently treating at least one youth (aged 3-21 years) using TF-CBT within the CBH clinic network were invited by email to participate, and if they chose to participate were asked a number of questions about their perspectives on telehealth [42]. Recruitment was carried out using a modified tailored design, which involved incorporating stakeholders into survey recruitment and providing a strong rationale for the utility of the survey data for clinicians and clients [43]. Clinicians received an email 1 week before the survey was distributed and were sent reminder emails 1 and 3 weeks after distribution. The survey was hosted on Qualtrics, a secure online service platform, and included an electronic informed consent form that stated that the survey would take approximately 30-40 minutes to complete. The survey was tested for usability on both computer and cellular platforms prior to distributing it to clinicians. Items appeared in the same order for each participant and all participants received the same questions. Survey items were distributed across multiple screens to decrease the number of questions per page and increase usability. At any time prior to submitting their responses, respondents could return to any previous screen and adjust their responses. To maintain anonymity, neither cookies nor IP addresses were used to identify duplicate responders, although upon revision of the completed surveys, there was no indication of duplicate survey entries by a single user. Clinicians completed the survey in July and August of 2020 and received a US $25 gift card for participating.

### Measures

#### Perspectives on Telehealth

Clinicians’ ratings of telehealth were measured using a survey instrument developed by Becevic et al [44] measuring clinician satisfaction with telehealth, and through several short-answer open-ended questions added by the authors. The Provider Survey was developed based on a literature review and the analysis of the role of the provider in telehealth delivery, which included 12 items: the first item asked clinicians whether or not they use telehealth, and the following 11 items asked about the extent to which they agreed with statements about telehealth on a 5-point Likert scale ranging from 1 (strongly agree) to 5 (strongly disagree). In responding to the survey questions, clinicians were instructed to consider any telehealth sessions they were conducting with clients via phone or video conference. In our sample, internal consistency of the scale was high (α=.89). Following the 12 items, we included 4 open-ended questions that assessed clinicians’ perspectives on how telehealth compares to in-person therapy (“What differences have you noticed in how you deliver treatment via telehealth compared to in-person?”), barriers to delivering treatment via telehealth (“Please report your top two barriers to delivering treatment via telehealth [ie, things that make it hard to deliver treatment via telehealth]”), facilitators to delivering treatment via telehealth (“Please report your top two facilitators to delivering treatment via telehealth [ie, things that make delivering telehealth easier]”), and how they could be supported in utilizing telehealth (“How can the PACTS team support you in delivering treatment via telehealth?”). Response rates to the 4 open-ended questions were high (range 36-45 respondents per question; total of 164 responses across questions).

#### Demographics

Clinicians completed a brief demographic questionnaire that included questions about age, gender, race, licensure status, years at current organization, and number of clients. This questionnaire also asked clinicians whether they were a salaried full-time worker or an independent contractor (ie, clinicians who contract with an organization but are paid per session rather than salaried).

### Analysis Plan

#### Quantitative Data

Demographic data were analyzed using descriptive statistics. Survey items were analyzed individually as well as by using a mean score of the 11 items. We also carried out exploratory posthoc correlation analyses to examine relationships among the mean score and the clinicians’ age, employment status, and number of clients.

#### Qualitative Data

Open-ended responses were analyzed using thematic analysis based on best practices and guiding principles in qualitative coding, and involved categorization of responses and coding of response frequency [45-47]. Content from open-ended responses was imported into Microsoft Excel (version 16.55), with one response per cell, and was first mapped onto different categories of repeating ideas that were derived from the data that were then formed into subthemes. Each response was coded using an open coding procedure; there were no predetermined categories and more than one code could be applied if warranted. Through an iterative process, each of the subthemes were then grouped into larger themes, and themes were grouped into domains. Frequencies were calculated for each domain. Following recommended qualitative research practices, a second coder coded 20% of the data and percent agreement was calculated using the total number of agreements divided by the total number of possible agreements [48]. Percent agreement was very high (96%) and the two coders reviewed and discussed discrepant codes until consensus was reached.

### Reporting

We use the Checklist for Reporting Results of Internet E-Surveys (CHERRIES) to guide our reporting of survey characteristics and results [49].

## Results

### Sample Characteristics

We received responses from 67 of the 198 clinicians contacted (response rate 34%); the 18 surveys with less than 50% of the items complete were not included, leaving 49 respondents (completion rate 25%). Although we reviewed timestamps of survey responses, responses were not eliminated due to long timestamps as clinicians were not instructed to complete the survey in a single sitting. Four of the remaining 49 respondents had never used telehealth, leaving a total of 45 clinicians (including staff with multiple clinical, supervisory, and administrative roles) in the study. These respondents were predominantly female (82%), master’s-level (93%) clinicians in their mid-30s (mean age 36 years); see Table 1 for detailed clinician demographics. They worked at 15 different agencies in Philadelphia (one agency had seven survey respondents, two had five respondents, two had four respondents, and the rest had three or less).

**Table 1:** Clinician demographics.

Characteristic		Respondents, n	Value
Age (years), mean (SD), range		42	36 (10), 25-64
**Gender, n (%)^a^**		44	
	Female		37 (82)
	Male		6 (13)
	Chose not to disclose		1 (2)
**Race, n (%)**		44	
	White		35 (78)
	Black		4 (9)
	Asian		2 (4)
	Mixed race or other race		2 (4)
	Chose not to disclose		1 (2)
**Ethnicity, n (%)**		44	
	Not Latinx		38 (84)
	Latinx		3 (7)
	Chose not to disclose		3 (7)
**Position type, n (%)**		44	
	Master’s-level clinician		25 (56)
	Social worker		8 (18)
	Other position		8 (18)
	Marriage and family clinician		2 (4)
	Psychologist		1 (2)
In a role with a supervisory or administrative component		45	18 (40)
**Employment status, n (%)**		45	
	Salaried full-time		28 (62)
	Independent contractor/fee-for-service		16 (36)
	Other		1 (2)
**Highest degree completed, n (%)**		44	
	Master’s degree		42 (93)
	Doctoral degree		2 (4)
**Licensure status, n (%)**			
	Licensed		23 (51)
	Not licensed		12 (27)
	In process		9 (20)
Clients seen per week, mean (SD), range		45	14 (8), 1-30
Hours worked per week across all jobs, mean (SD), range		45	39 (11), 5-60
Years of experience in full-time human services work, mean (SD), range		44	10 (8), 2-30
Years of experience in role of clinician, mean (SD), range		44	9 (8), 1-30
Years worked at present agency, mean (SD), range		44	5 (4), 1-16

^a^Percentages were calculated using a denominator of the 45 clinicians who responded.

### Quantitative Results

All 45 clinicians completed each of the telehealth survey items; there were no missing data (see Table 2). Across items, clinicians tended to agree with the statements presented in the survey, with mean responses falling between “neutral” and “agree” on 7 (64%) items. Clinicians agreed more strongly with two statements in particular: “Telehealth helps me to converse with my clients” (mean 2.0, SD 0.7) and “For the moment, I am satisfied with the work I’ve done through telehealth” (mean 2.0, SD 0.6). Clinicians also responded with more neutrality to one item (“The images and sounds of telehealth gear are clear and crisp”; mean 3.0, SD 1.0) and more disagreement to one item (“I prefer telehealth visits over visits that are in person”; mean 3.8, SD 1.0). Posthoc exploratory correlational analyses did not suggest a relationship between clinician telehealth ratings and their age or number of clients. However, clinician employment type appeared to be associated with the mean telehealth score, with independent contractors rating telehealth more positively compared to full-time salaried employees (B=–0.32, 95% CI –0.47 to –0.02; *P*=.03).

**Table 2:** Clinicians’ ratings of telehealth (N=45)^a^.

Survey item	Mean (SD)	Mode (range)
It is easy to run and use the telehealth system	2.4 (0.9)	2 (1-4)
I am confident and feel at ease when I use the telehealth system	2.5 (1.0)	2 (1-5)
Telehealth gives me the chance to build and keep a personal bond with each of my clients	2.3 (0.9)	2 (1-5)
Telehealth fits well with each day’s workflow	2.3 (0.8)	2 (1-4)
The images and sounds of telehealth gear are clear and crisp	3.0 (1.0)	3 (1-5)
I get more done in my day when I see clients through telehealth	2.8 (1.1)	3 and 4 (1-5)
Telehealth helps me to converse with my clients	2.0 (0.7)	2 (1-4)
Telehealth allows me to see more clients	2.4 (1.1)	1 and 3 (1-4)
I am able to treat my clients’ needs well through telehealth	2.4 (0.8)	2 (1-4)
I prefer telehealth visits over visits that are in person	3.8 (1.0)	4 (1-5)
For the moment, I am satisfied with the work I’ve done through telehealth	2.0 (0.6)	2 (1-4)
Mean score	2.5 (0.6)	2.5 (1.1-3.8)

^a^1=strongly agree, 2=agree, 3=neutral, 4=disagree, and 5=strongly disagree.

### Qualitative Results

We developed five domains based on the data, each comprised of multiple themes and, within those themes, subthemes (see Table 3 for domains, themes, and subthemes; see Multimedia Appendix 1 for additional examples). The domain most frequently mentioned by clinicians (n=68 responses) was changes to therapy process and content, which involved clinicians’ observations about the ways in which telehealth has changed the process of carrying out therapy (ie, therapy process) as well as the nature of the therapy content itself (ie, therapy content). This domain included observations about the pros and cons to the virtual therapy process. Some clinicians noted that teletherapy offers an opportunity for more creativity and collaboration than in-person therapy, such as in jointly coming up with realistic plans for coping based on the clients’ home environment. However, they also noted risks associated with the shift to telehealth, including no longer sharing physical space with clients and a lack of nonverbal communication. Some clinicians also noted ways in which delivering treatment over telehealth appears to be slowing the pace of therapy. Clinicians also noted the need for adaptation of and modification to the therapeutic intervention being used. They also noted additional challenges, including difficulty with implementing core TF-CBT practice elements over telehealth (eg, trauma narrative), limitations in the use of physical tools and supplies that they typically rely upon (eg, games and physical books), changes to the content of their sessions and the aspects most emphasized (eg, less emphasis on mindfulness), and changes to the format of therapy (eg, shorter sessions).

Clinicians’ responses also frequently fell into the domain of engagement, retention, and attendance (n=59 responses). Many clinicians noted changes in attendance and retention associated with the shift to telehealth, with most identifying an increase in attendance and retention, and greater ease of scheduling (although a few cited a decrease in attendance and retention with certain clients). Clinicians also noted differential engagement challenges across clients. Although across the board there were widespread challenges with engagement, some clients were particularly hard to engage (especially young children), whereas in select cases, clients were more engaged (eg, some teenagers, some children with higher anxiety). Additionally, respondents observed an increase in caregiver engagement, including more consistent involvement of caregivers in therapy and specific ways in which caregivers impacted client engagement.

We developed a third domain around technology (n=57 responses). Clinicians noted the indispensability of access to technological devices, the frequent problem of internet access, and the presence of digital disparities. Clinicians noted that it is essential that clients and clinicians have the devices needed to access telehealth, such as a computer with a webcam, and the platforms needed to carry out telehealth visits. Clinicians also noted the frequency of internet connectivity issues and the ways in which these issues have an adverse effect on therapy. They also highlighted digital disparities that negatively impact low-income clients; in the context of telehealth, lack of access to crucial resources may mean inability to access mental health services altogether.

The importance of training, resources, and support was identified as a fourth domain (n=52 responses). Many clinicians highlighted the need for more guidance pertaining to telehealth, including didactic trainings and supervision and consultation. Clinicians also noted the need for resources and funding specific to telehealth, including provision of telehealth-compatible physical supplies, telehealth-specific funding and incentives, and provision of technological devices and internet access, particularly to low-income clients. Many also highlighted the value of shared telehealth information and tools through their organization or via email, including recommendations for creative online resources, distribution of telehealth tips between clinicians, and online materials shared with clients.

Finally, clinicians’ responses highlighted a fifth domain pertaining to barriers and facilitators to telehealth (n=43 responses). Clinicians noted that the individual characteristics of clinicians, clients, and caregivers can serve to facilitate or hinder successful therapy. For example, clinicians who are creative and flexible may have an easier time implementing treatment over telehealth, as will those with greater motivation and greater bandwidth. Additionally, caregiver support of clients is impactful in facilitating success. Many responses also highlighted drawbacks and limitations of telehealth; even in the best of circumstances, telehealth increases certain burdens on clinicians and poses logistical limitations, leaving some clinicians feeling exhausted. Finally, telehealth is more acceptable to some clients and clinicians than to others. Many clients find telehealth to be convenient, although some may find it more challenging and uncomfortable. Additionally, clinicians suggested that the acceptability of telehealth is not a constant and may increase as time passes.

**Table 3:** Domains, themes, subthemes, and examples^a^.

Domains, themes, and subthemes				Examples
**Changes to therapy process and content**				
	**Altered therapy process has pros and cons**			
		Loss of shared physical space	Lack of in-person contact feels different	
		Hindered nonverbal communication	Can’t read body language or body cues	
		Opportunity for creativity, collaboration, and cooperative planning	Easier identification of realistic plans for coping	
		Potential effect on pace of therapy	Progress moving slower	
	**Need for adaptation and modification of content for telehealth**			
		Difficulty implementing core practice elements	Challenging to get child input in trauma narrative	
		Inability to use preferred tools and supplies	The therapy tools feasible over telehealth are less engaging	
		Changes in therapy content and technique	Using more visuals, discussion questions, and planned activities	
		Changes in therapy process, format, and structure	Clients want check-ins and not full sessions	
**Engagement, retention, and attendance**				
	**Changes in attendance and retention**			
		General increase in attendance and retention	Decreased no-shows	
		Greater scheduling flexibility	Easier to reschedule if needed	
		Occasional negative impact on attendance and retention	Some clients are forgetful and need more reminders	
	**Differential engagement challenges across clients**			
		Widespread challenges with engagement across many clients	Difficulty paying attention	
		Some groups particularly hard to engage	Particular difficulty engaging young clients	
		Small subset have increased engagement	Some clients able to open up more over telehealth compared to in person	
	**Caregiver engagement and involvement has generally increased**			
		More direct and consistent contact with caregivers	Easier for caregivers to be involved	
		Caregivers can aid in client engagement	Caregivers can increase buy-	
**Technology**				
	**Access to and facility with technological devices and platforms is crucial**			
		Need for appropriate devices and accessories	Both clients and clinicians require devices with video capabilities	
		Need for access to specific programs and capabilities	Access to HIPAA^b^-compliant video platforms (preferably with paid subscription) is essential	
	**Internet access is a problem**			
		Many clients have connectivity issues	Access to stable internet not always available	
		Connectivity issues have adverse effect on therapy	Poor network connections (frequent glitches, bad lags) and internet interruptions (calls dropping in middle of session) are disruptive to therapy	
	**Digital disparities are undeniable**			
		Technological issues disproportionately affect low-income clients	Access to technology limited among low-income clients	
		Those without access to telehealth may be unable to receive care	Without stable internet or phone connection, telehealth becomes inaccessible	
**Training, resources, and support**				
	**Clinicians want more training and support in telehealth**			
		Clinicians want didactic trainings	Desire for webinars	
		Need for supervision and consultation	Support from colleagues and employer makes a difference	
	**Provision of resources, funding, and incentives is needed**			
		Physical supplies can still be used	Physical items can be sent to clients	
		Need for funding and incentives	Clinicians and clients can benefit from funds allocated toward supplies	
		Technological devices and internet access is a must	Clients need access to appropriate devices for telehealth	
	**Desire for continued sharing of information, suggestions, and tools for telehealth**			
		Use online resources creatively	Make use of websites, apps, worksheets, books	
		Distribute telehealth tips between clinicians	Tips for how to support (young) children and parents using telehealth	
		Share materials with clients	Provide clients with interactive materials and worksheets	
**Differential barriers, facilitators, and acceptability across clinicians and clients**				
	**Individual characteristics of clinicians, clients, and caregivers can facilitate or hinder successful therapy**			
		Creativity and flexibility are key	Clinician and client creativity are key to successful telehealth use	
		Motivation matters for clinicians, caregivers, and clients	High motivation and drive from client and family facilitates success	
		Logistics and bandwidth make a difference	Clinicians who have time to prepare in advance may find telehealth easier	
		Caregiver support and involvement in therapy is a huge facilitator	Caregivers can create a safe space for therapy	
	**Telehealth has drawbacks and limitations**			
		Telehealth increases burden on clinicians	Increased preparation and planning required	
		Limitations exists even when done well	Many tangible tools and games cannot be used	
	**Differential acceptability of telehealth across clients and clinicians**			
		Increased convenience and comfort for some clients and clinicians	Many clients and families find telehealth to be more convenient	
		Telehealth can be challenging and uncomfortable	Telehealth feels limited to many clinicians	
		Acceptability may change over time	Getting easier over time and clinicians getting better and more confident	

^a^Additional examples of each subtheme can be found in Multimedia Appendix 1.

^b^HIPAA: Health Insurance Portability and Accountability Act.

## Discussion

### Principal Findings

The perspectives shared by the clinicians in our sample highlight an array of insights that may reveal common challenges and benefits to EBP delivery via telehealth in CMH settings. They also point to the next steps for practice and directions for future research. Clinicians observed that telehealth has pros and cons, and may better fit some clients than others. They also made clear the distinction between access to telehealth and engagement in telehealth; while session attendance is important, it is only half the battle. In order for clients to benefit from therapy, they must also be engaged. Finally, although telehealth may be a more convenient option for families who can access it, there are many clients, particularly those from low-income families, for whom it is not available due to lack of internet access or lack of privacy, among other reasons. Given that TF-CBT is widely implemented on a national level and that the challenges to implementing TF-CBT in our sample may be similar to challenges that arise in implementing EBPs more broadly in other CMH settings, these findings may be useful in informing and facilitating implementation of EBPs across CMH settings nationally. Further, it is likely that the barriers faced by clinicians in our sample reflect those faced by clinicians in other CMH settings where resources are limited, and caseloads and administrative burdens are relatively high. Suggestions made by clinicians in our sample may be useful in supporting clinicians in other CMH settings in delivering EBPs over telehealth.

### Telehealth May Be a Better Fit for Some Clients and Clinicians Than for Others

Our data suggest that clinicians do not view telehealth as a one-size-fits-all modality for treatment delivery. Clinicians broadly reported that telehealth can be a good option for many clients in that it is convenient, generally acceptable, and may even provide surprising benefits such as increased creativity and collaboration, increased parental involvement, and an ability for clinicians to gain a more intimate understanding of their clients’ home environment (ie, more ecological validity), which can provide clinically important information that would not otherwise be available. These benefits are supported by other studies [50,51], and provide promising implications regarding the potential for the longstanding use of telehealth for clients who prefer this modality. Positive views about telehealth are also reflected in our quantitative data, which show relatively favorable ratings of telehealth, especially when considering the quick pivot and lack of preparation. It is unclear, however, whether these perspectives would be held outside of the context of a pandemic or if clinicians are merely “finding a silver lining” in the midst of an otherwise difficult set of circumstances. Future research should investigate how acceptable and satisfactory telehealth-delivered EBPs are to clinicians and clients postpandemic. It may be that there is differential effectiveness of telehealth wherein individuals who find it more acceptable have better therapy outcomes than those who find it less acceptable. Future research should address this question.

Despite these positive elements of telehealth, clinicians noted additional challenges to both clients and clinicians. Some clinicians reported feeling more burdened and exhausted than when delivering therapy in person. Although clinicians tended to agree that telehealth is a good solution “for the moment,” the survey item with the greatest disagreement was “I prefer telehealth visits over visits that are in person.” It seems that clinicians may be accepting telehealth as a short-term solution without necessarily preferring it. This contrasts with findings within a sample of clinicians at a nonprofit hospital system serving predominantly privately insured clients, as roughly half indicated that they would like to continue using telehealth after the pandemic [37]. It may be that utilization of telehealth in a CMH clinic puts higher burdens on clinicians than in higher-resourced systems. More work is needed to identify how to best support CMH clinicians in delivering treatment via telehealth. It is also possible that there are individual differences across clinicians that determine the acceptability of telehealth, including differences in personality or differences in job characteristics. For example, in our sample, clinicians who were independent contractors rated telehealth more highly than did full-time salaried clinicians. We hypothesize that for independent contractors, the reduction in transportation time may make a greater difference than for salaried clinicians, particularly if they are accustomed to traveling between agencies. It may also be that the general flexibility and variability in independent contractor positions prepared these clinicians well to adapt to a novel situation. Future work harnessing their insights may be beneficial in identifying further ways to improve telehealth and to optimize the experience of salaried full-time workers.

Telehealth raises a number of challenges that may not be mutable even in the best of circumstances, such as the lack of confidentiality in households without a private space, the inability to read body language, and the fact that some clients may simply be too young to engage in telehealth. Future research should examine the question of what aspects of treatment can be best carried out using telehealth, and whether, analogous to the question of what forms of psychotherapy work best for which clients, we can gain greater insight into “personalized telehealth” and identify for whom telehealth is best suited [52,53]. It may be that at least some of the challenges highlighted in these data can be overcome through provision of supplies and through dissemination of information. This can come in various forms, including didactic webinars, shared resources, and consultation and supervision, all of which the clinicians in our sample highlighted as a desired form of support. For example, our system sponsored a webinar led by a clinical director at one of the agencies highlighting creative ways in which to enact telehealth. The APA has compiled a list of telehealth resources, including other webinars and information about regulatory guidelines. There are also TF-CBT–specific resources about telehealth, such as the Telehealth Outreach Program [54] and the National Therapist Certification Program [55] from the Medical University of South Carolina. Similar telehealth resources for clinicians are available for a broad array of treatments and disorders. Previous work has also examined the ways in which training in delivering treatment via telehealth may be integrated into graduate training, which may be a proactive way to prepare future clinicians [56].

The challenges that clinicians reported with regard to burden may be due to the abrupt shift to telehealth and stage of maturation when clinicians were surveyed. Clinicians were surveyed within the first 6 months of the shift to telehealth, and their perspectives may have changed with further experience. Indeed, there is evidence to support the notion that telehealth gets easier with time. A systematic review of clinician attitudes toward telehealth revealed that those who had more experience with the modality felt more positively about it than those who did not use it or were new to it [57].

The extent to which telehealth continues to be utilized in the future will depend not only on how acceptable it is to clients and clinicians but also on regulatory factors such as reimbursement from payors and licensure restrictions [58]. Although it is unclear whether the temporary allowances made in the context of a global pandemic will continue, there is promising data to suggest that this time of increased telehealth use has resulted in a ramp-up in telehealth infrastructure and a decrease in some of the barriers that existed previously [59]. It may be that with sufficient interest and attention given to strengthening telehealth systems, and with research to support effectiveness of its use, sufficient pressure will be put on policymakers and payors to make this modality a sustainable long-term option.

### Attendance Is Necessary but Not Sufficient

Important questions remain with regard to engagement and parental involvement. Telehealth may reduce barriers in accessing care, such as transportation barriers, and our findings are consistent with previous work that noted less attrition with depressed clients when using telehealth [35,60,61]. Factoring in clients’ busy schedules as well as the technological advances and relative accessibility of internet connection, telehealth may make it easier for many clients to attend sessions regularly. However, for these clients who find telehealth more convenient, the extent to which they successfully engaged in sessions remains unclear. Many clinicians noted that engagement in sessions remains a challenge, particularly for younger children. Future research should compare client attendance, engagement, and outcomes of in-person versus telehealth-delivered therapy sessions to better understand how telehealth influences these factors. Despite challenges with client engagement, there was unanimous agreement among the clinicians who commented on parental engagement that telehealth facilitates involvement of parents. Clinicians indicated that when parents are involved, therapy seems to go better. Even if telehealth does not remain the first-line mode of treatment, it may be worth investigating whether, even within the context of the return to face-to-face therapy with clients, parents can continue to engage virtually.

### Digital Disparities Must Be Addressed

Even if the shift to telehealth increases access for some clients, attention must be paid to digital disparities. Clinicians noted the ways in which digital disparities create issues with access to therapy, and that among the clients who can access therapy, a subset may have impaired quality of connectivity. Disruptions in connectivity are not conducive to therapeutic progress, and in many cases make it more difficult to foster a strong therapeutic alliance. This was reflected in the clinicians’ relatively low agreement on the quantitative survey that telehealth is “clean and crisp.” Although families that are in a low-income category may benefit, in theory, from the elimination of transportation barriers in accessing care, if they do not have reliable internet access, then telehealth is not a solution. It is important to note that the challenges associated with digital disparities are rooted in far wider–reaching issues of inequity that must be addressed for longstanding change to occur. Recent work has focused on mitigating digital disparities through the lens of health equity. Future implementation of telehealth should be guided by consideration of how to reduce digital disparities across the individual, institutional, and broader social levels [62]. In the interim, there may be creative solutions to help reduce these disparities within existing systems, including allowing clients without home internet access to engage in therapy at school. Additional short-term solutions include leveraging auxiliary staff or community health workers to aid families in accessing telehealth, or in providing families with temporary devices and internet access to engage in care, as was successfully demonstrated in one study [13,63,64].

### Limitations

This study has several limitations. First, and most importantly, the shift to telehealth occurred within the context of a global pandemic, and therefore the generalizability of these observations to more typical circumstances is unknown. Second, the clinicians all belong to one public mental health system, and the extent to which their perspectives can be generalized to other settings is unclear. For example, there may be idiosyncrasies to the Philadelphia youth community behavioral health system that are unique, and the perspectives of these clinicians may not capture challenges in other settings such as rural areas. Of note, a recent study of clinician attitudes toward telehealth in a rural CMH setting found that clinicians’ concerns were similar to those of clinicians in our sample [65]. Third, although our sample included therapists from a diverse set of agencies, some agencies were more represented than others and we did not account for nesting in our analyses due to power limitations. Fourth, the response rate to the survey was low (although typical for online surveys), and the sample may be biased toward clinicians who were functioning more highly and thus not representative of the broader sample of clinicians invited to participate [66,67]. However, demographic characteristics of the clinicians in our sample were reflective of those of the broader public mental health workforce of Philadelphia, providing some support for generalizability [68]. Fifth, although our intention was to understand clinicians’ perspectives and we see self-report as a strength, there are potential response biases, blind spots, and limitations associated with self-reporting. Although the anonymity of a survey may serve to reduce certain types of response biases, the data are not as extensive as those obtained with an in-depth interview. Nonetheless, we found a lot of overlap in clinicians’ responses, suggesting that theoretical saturation had been reached. Sixth, the Provider Survey used to capture clinicians’ ratings of telehealth has not yet been validated. We selected this measure due to the relative death of surveys on provider perspectives on telehealth; thus, we opted to use a published measure rather than creating our own. Finally, this study does not include client and family perspectives, and future qualitative work with these groups will be important, not only in understanding how much they like or dislike telehealth but also in allowing their views to help shape future developments in telehealth systems to increase acceptability.

### Conclusion

This study examined the perspectives of clinicians working in a public mental health system providing TF-CBT via telehealth. Our findings lend insight into the challenges and benefits associated with delivering EBP via telehealth in the context of a system that shifted rapidly into this new delivery modality. Future work should determine which clinicians and clients are best suited for telehealth, identify how to better engage clients, and reduce digital disparities. The future of telehealth delivery within public mental health systems will depend largely on reimbursement streams, and the extent to which these services will be utilized more permanently may be revealed once it becomes safe to resume in-person therapy. Although the long-term trajectory of telehealth is largely unknown and our findings are derived from a single sample of clinicians, there is likely some universality to their observations, particularly in urban CMH settings. The insights and recommendations of the clinicians in our sample may help to inform future research and strengthen telehealth services for youth in need of mental health care.

## Appendix

Multimedia Appendix 1 Domains, themes, and subthemes with additional examples.

## Abbreviations

APA: American Psychological Association
CBH: Community Behavioral Health
CHERRIES: Checklist for Reporting Results of Internet E-Surveys
CMH: community mental health
DBHIDS: Department of Behavioral Health and Intellectual Disability Services
EBP: evidence-based practice
OCD: obsessive-compulsive disorder
PACTS: Philadelphia Alliance for Child Trauma Services
PTSD: posttraumatic stress disorder
TF-CBT: trauma-focused cognitive behavioral therapy
