# Supplementary material for: Community Mental Health Clinicians’ Perspectives on Telehealth During the COVID-19 Pandemic: Mixed Methods Study
Source: JMIR Pediatr Parent. 2022 Mar 3;5(1):e29250. doi: 10.2196/29250 (PMC8989386; doi:10.2196/29250)
Supplement: Multimedia Appendix 1 [file pediatrics_v5i1e29250_app1.docx]

Multimedia Appendix 1. Domains, themes, subthemes, and extended examples.

| Domains | Themes | Sub-themes | Examples |
| --- | --- | --- | --- |
|  |  |  |  |
| **Changes to therapy process and content** |  |  |  |
|  | Altered therapy process has pros and cons | Loss of shared physical space | Lack of in-person contact feels different; lack of access to physical supplies; lack of client privacy; difficulty building rapport; can’t use preferred tools and strategies; difficulty with spontaneity and emotional connection |
|  |  | Hindered non-verbal communication | Can’t read body language or body cues; “a lot that is missing” |
|  |  | Opportunity for creativity, collaboration, and cooperative planning | Easier identification of realistic plans for coping; helpful to see clients in home environment; safer for client and clinician to use telehealth during pandemic |
|  |  | Potential effect on pace of therapy | Progress moving slower; sense that “much less work is being done”; feeling less connected |
|  | Need for adaptation and modification of content for telehealth | Difficulty implementing core practice elements | Challenging to get child input in trauma narrative; difficult to practice techniques like mindfulness and deep breathing |
|  |  | Inability to use preferred tools and supplies | The therapy tools feasible over telehealth are less engaging; need for creation of telehealth-specific resources for TF-CBT |
|  |  | Changes in therapy content and technique | Using more visuals, discussion questions and planned activities; using telehealth contract at start of therapy |
|  |  | Changes in therapy process, format, and structure | Clients want check-ins and not full sessions; sessions being conducted only with caregivers when clients are too young for telehealth |
| **Engagement, retention, and attendance** |  |  |  |
|  | Changes in attendance and retention | General increase in attendance and retention | Decreased no-shows; decreased transportation barriers |
|  |  | Greater scheduling flexibility | Easier to reschedule if needed; clinician has more availability throughout day |
|  |  | Occasional negative impact on attendance and retention | Some clients are forgetful and need more reminders; have had to discharge some clients due to technology issues or lack of safe space for therapy |
|  | Differential engagement challenges across clients | Widespread challenges with engagement across many clients | Difficulty paying attention; not taking therapy seriously; throwing phone around; taking therapy call in inappropriate situations (e.g.. at grocery store or while cooking dinner) |
|  |  | Some groups particularly hard to engage | Particular difficulty engaging young clients; harder to establish rapport with clients “met” over telehealth; some teens prefer just phone |
|  |  | Small subset have increased engagement | Some clients able to open up more over telehealth compared to in person |
|  | Caregiver engagement and involvement has generally increased | More direct and consistent contact with caregivers | Easier for caregivers to be involved; able to reach caregivers who might otherwise not be able to engage |
|  |  | Caregivers can aid in client engagement | Caregivers can increase buy-in; caregivers can help get clients set up for therapy |
| **Technology** |  |  |  |
|  | Access to and facility with technological devices and platforms is crucial | Need for appropriate devices and accessories | Both clients and clinicians require devices with video capabilities; headphones are helpful for privacy; some clients have devices provided by school which are helpful unless they have restricted access; need for technological literacy and familiarity with software |
|  |  | Need for access to specific programs and capabilities | Access to HIPAA-compliant video platforms (preferably with paid subscription) is essential; screen sharing capability is crucial to successful teletherapy; applications such as Microsoft Paint can be helpful tools |
|  | Internet access is a problem | Many clients have connectivity issues | Access to stable internet not always available; client phones may be disconnected; need for improvement in client connectivity |
|  |  | Connectivity issues have adverse effect on therapy | Poor network connections (frequent glitches, bad lags) and internet interruptions (calls dropping in middle of session) are disruptive to therapy; may impair smooth flow of session and in some cases even result in having to restart or end session; inability to connect on some days results in missed sessions |
|  | Digital disparities are undeniable | Technological issues disproportionately affect low-income clients | Access to technology limited among low-income clients; engagement and attendance likely affected by technological limitations |
|  |  | Those without access to telehealth may be unable to receive care | Without stable internet or phone connection, telehealth becomes inaccessible |
| **Training, resources, and support** |  |  |  |
|  | Clinicians want more training and support in telehealth | Clinicians want didactic trainings | Desire for webinars; particular interest in how to carry out telehealth with young children; training on how to use telehealth platforms |
|  |  | Need for supervision and consultation | Support from colleagues and employer makes a difference; helpful ideas shared via consultation with other clinicians |
|  | Provision of resources, funding, and incentives is needed | Physical supplies can still be used | Physical items can be sent to clients; books and manuals (including electronic versions) continue to be useful |
|  |  | Need for funding and incentives | Clinicians and clients can benefit from funds allocated toward supplies; incentives for families to attend therapy can be effective |
|  |  | Technological devices and internet access is a must | Clients need access to appropriate devices for telehealth; clients need support in accessing stable internet; could provide clients with mobile hotspots |
|  | Desire for continued sharing of information, suggestions, and tools for telehealth | Use online resources creatively | Make use of websites, apps, worksheets, books; participate in telehealth groups that share information |
|  |  | Distribute telehealth tips between clinicians | Tips for how to support (young) children and parents using telehealth; distribute suggestions about creative ideas in implementing TF-CBT through telehealth |
|  |  | Share materials with clients | Provide clients with interactive materials and worksheets |
| **Differential barriers, facilitators, and acceptability across clinicians and clients** |  |  |  |
|  | Individual characteristics of clinicians, clients, and caregivers can facilitate or hinder successful therapy | Creativity and flexibility are key | Clinician and client creativity are key to successful telehealth use; important to meet clients where they are at |
|  |  | Motivation matters for clinicians, caregivers, and clients | High motivation and drive from client and family facilitates success; low motivation from clinicians is a barrier |
|  |  | Logistics and bandwidth make a difference | Clinicians who have time to prepare in advance may find telehealth easier; clinicians with own children at home may have greater challenges |
|  |  | Caregiver support and involvement in therapy is a huge facilitator | Caregivers can create a safe space for therapy; can talk to clients about what they are doing in therapy |
|  | Telehealth has drawbacks and limitations | Telehealth increases burden on clinicians | Increased preparation and planning required; telehealth is exhausting and causes eye strain and fatigue from tech; emotional boundary challenges in bringing therapy into home space; more effort required to keep conversation going; inadequate preparation for shift to telehealth; easier to deliver services in person |
|  |  | Limitations exists even when done well | Many tangible tools and games cannot be used; limitations to working with young children even in best of circumstances; clinicians cannot respond to crises in same manner they would in person |
|  | Differential acceptability of telehealth across clients and clinicians | Increased convenience and comfort for some clients and clinicians | Many clients and families find telehealth to be more convenient; some clinicians enjoy working from home; some teens and anxious clients prefer doing therapy from home; less stress for clients and parents |
|  |  | Telehealth can be challenging and uncomfortable | Telehealth feels limited to many clinicians; many clients feel uncomfortable; switch to telehealth seems to have greatest impact on young clients |
|  |  | Acceptability may change over time | Getting easier over time and clinicians getting better and more confident; need for normalization of telehealth services to facilitate widespread habituation to their use; important to demonstrate positive impact to increase buy-in from administrators |
